# Looks like home: numerosity, but not spatial frequency guides preference in zebrafish larvae (*Danio rerio*)

**DOI:** 10.1007/s10071-024-01888-0

**Published:** 2024-07-27

**Authors:** Elisabeth Adam, Mirko Zanon, Andrea Messina, Giorgio Vallortigara

**Affiliations:** CIMeC - Center for Mind/Brain Sciences, University of Trento, 38068 Rovereto, Italy

**Keywords:** Zebrafish, Numerosity, Spatial frequency, Counting, Choice, Habitat selection

## Abstract

Despite their young age, zebrafish larvae have a well-developed visual system and can distinguish between different visual stimuli. First, we investigated if the first visual surroundings the larvae experience during the first days after hatching shape their habitat preference. Indeed, these animals seem to “imprint” on the first surroundings they see and select visual stimuli accordingly at 7 days post fertilization (dpf). In particular, if zebrafish larvae experience a bar background just after hatching, they later on prefer bars over white stimuli, and vice versa. We then used this acquired preference for bars to investigate innate numerical abilities. We wanted to specifically test if the zebrafish larvae show real numerical abilities or if they rely on a lower-level mechanism—i.e. spatial frequency—to discriminate between two different numerosities. When we matched the spatial frequency in stimuli with different numbers of bars, the larvae reliably selected the higher numerosity. A previous study has ruled out that 7 dpf zebrafish larvae use convex hull, cumulative surface area and density to choose between two numerosities. Therefore, our results indicate that zebrafish larvae rely on real numerical abilities rather than other cues, including spatial frequency, when spontaneously comparing two sets with different numbers of bars.

**Supplementary Information:**

The online version contains supplementary material available at 10.1007/s10071-024-01888-0.

## Introduction

The zebrafish *Danio rerio* inhabits freshwater bodies across the Indian subcontinent where it is found in a wide range of habitats from fast flowing streams to shallow clear ponds and rice fields with dense vegetation (Arunachalam et al. 2013; Spence et al. 2006, 2008; Sundin et al. 2019). This small fish generally breeds between April and October, with the female choosing aerated substrates as spawning site (Spence et al. 2007; Sundin et al. 2019). Once the larvae hatch, they use vertical surfaces, rocks, sandy substrate or plants to reach the water surface to inflate their swim bladder (Lindsey et al. 2010). After about 3 days post fertilization (dpf) the zebrafish larvae respond to objects moving across their visual field (optokinetic response) and at around 5–7 dpf they are able to actively follow moving visual stimuli (optomotor response), swim and track prey (Fero et al. 2011; McElligott and O'Malley 2005; Neuhauss 2003).

The zebrafish eye generally takes about one month to develop to its adult form. The maturation in the first few days is, however, incredibly fast and at 5 dpf signal transmission from the photoreceptors to the second-order neurons is fully developed. At 6 dpf, the typical layering of vertebrate eyes is already established (Gestri et al. 2012). After hatching, vision is dominated by cones important for color perception and high-light-intensity vision (Bilotta et al. 2001), which allows zebrafish larvae rather sophisticated innate visual discrimination abilities. Zebrafish larvae can discriminate between objects with different colors, shapes, and orientations. When given a choice between horizontal and vertical bars, they spent significantly more time near vertical bars. Since vertical bars resemble vegetation and elongated horizontal bars possible predators, this innate preference could be a simple computational mechanism to avoid predation (Gatto et al. 2021).

Other (semi)aquatic species have also been shown to use vertical bars as proxy for a safe place to hide: In the sea, the brittle star *Ophiomastix wendtii* uses its basal vision to move towards cracks in the reef that protect it from predators such as wrasse, parrotfish and mojarra. When presented with a high-contrast vertical bar in the lab, the brittle star moves towards it to seek safety (Sumner-Rooney et al. 2021). The oyanirami fish *Coreoperca kawamebari* is known to change its coloration depending on its internal state. If it experiences fear of a predator or loses a fight with a conspecific, it shows a stripe pattern resembling the vertical vegetation of its habitat. The coloration change helps it to blend in with the plants to avoid being attacked by the predator or dominant animal. As zebrafish larvae, the oyanirami shows a preference for vertical bars, which resemble vertical water plants, over horizontal bars, which could be perceived as predators (Kohda and Watanabe 1986).

The numerosity of vertical bar stimuli can be used by animals to make a choice between two possible hiding places. Generally, a higher number of bars might indicate more vegetation and therefore a safer place to hide while less vegetation might indicate a less preferable hiding place. The Italian treefrog *Hyla intermedia* shows a preference for microhabitats with abundant and tall grass right after metamorphosis. When tested in a two-choice paradigm with different numbers of green vertical bars resembling patches of grass, it showed a preference for the larger numerosity for bar ratios up to 0.5 (Lucon-Xiccato et al. 2018).

More generally speaking, vertical bars are not only relevant in terms of environmental cues, but they seem to be fundamental markers even for sexual selection in different fish. For example, the sailfin molly (*Poecilia latipinna*) female prefers males with bars over males without bars and symmetrical (4 *vs*. 4 bars) over non-symmetrical males (3 *vs.* 5 bars). Symmetry is an honest signal in ornaments since asymmetry can be caused by environmental factors and indicate low genetic quality. If given a choice, the females therefore prefer the male with higher genetic quality (Schlüter et al. 1998). Swordtail fish females (*Xiphophorus cortezi*) prefer males with symmetrical vertical bars as well (Morris and Casey 1998). Interestingly, they seem to use bar frequency and convex hull as well as total pigmented area as proxies for male quality (Morris et al. 2001).

Zebrafish larvae have previously been shown to be able to discriminate between different numbers of bars resembling vertical vegetation (Gatto et al. 2021; Lucon-Xiccato et al. 2023). In their study, the authors tested different ratios of bars with increasing difficulty on zebrafish larvae (7 dpf) and matched the stimuli for overall surface area, convex hull, luminance and density. Still, the larvae showed a robust preference for the larger numerosity. Interestingly, habituation with a bar background during the development was necessary to spark their interest in bar stimuli, since zebrafish larvae habituated with a white background did not show any preference (Lucon-Xiccato et al. 2023). It is also known from earlier studies that zebrafish prefer the body coloration pattern of the social group they experience early on in life. If they are exposed to fish with stripes early on, they later prefer shoals with striped fish, while if they experience fish without stripes, they prefer shoals without stripes (Engeszer et al. 2004; Spence and Smith 2007). This raises the question whether habitat choice is similarly influenced by early life experience. That could be an especially interesting question given that zebrafish inhabit very diverse habitats in the wild, ranging from dense aquatic vegetation to open areas. Lucon-Xiccato et al. (2023) showed that zebrafish larvae habituated with a bar background choose bars over a white stimulus. The authors did not, however, see a clear preference for the white stimulus when habituating the larvae with a white background. We therefore, as first aim, tried to replicate their findings, this time by transferring the eggs before hatching to the pre-selected environment, so the larvae would already hatch experiencing either the bars or the white background. We also added an additional control during the test phase to exclude the white walls of the test tank as possible confounding factor. Altering the protocol in this way allowed us to show that zebrafish larvae imprint on the visual habitat they hatch in and prefer the habitat they experienced before.

When choosing a habitat or place to hide from a predator, the processing speed of the visual system and its underlying neuronal network could play an important role. Previous work has shown that the visual recognition and categorization of complex natural scenes can be very fast. This implies a rather simple mechanism of natural scene recognition since processing of complex information takes longer time. It has been shown that spatial frequency (SFQ) information of an image can contribute to this process. Low spatial frequencies are associated with coarse information such as the global shape of an object. High spatial frequencies are important for the transmission of fine details such as edges and borders of an object (Kauffmann et al. 2014). While it is well known that different continuous variables can be used by animals to discriminate between numerosities (Leibovich et al. 2017), hardly any studies have taken a look at the role of spatial frequency in number cognition, yet. The studies that have been published have found quite different results. Simpler organisms like honeybees seem to rely on spatial frequency when discriminating between stimuli with different numerosities (MaBouDi et al. 2021). Adult archerfish and humans have instead been shown to rely on numerosity rather than spatial frequency in number cognition tasks (Adriano et al. 2021; Potrich et al. 2022).

In this study, we were interested in examining whether juvenile vertebrates already show real numerical abilities or whether they rely on simpler mechanisms (i.e., spatial frequency) during their development. Zebrafish larvae offer a great model system to investigate numerical abilities in juvenile animals since they still develop after hatching and can be tested shortly after they hatch. As a second aim, we specifically investigated whether zebrafish larvae use real numerical abilities or spatial frequency information to choose between different numbers of bars. First, we created a baseline testing larvae with different numbers of bars without altering the spatial frequency information. Then we matched the spatial frequency across stimuli (by equalizing their amplitude power spectrum) while comparing different numerosities. This allowed us to pinpoint whether zebrafish larvae use numerical or spatial frequency information of a stimulus to choose between two numerosities.

## Material and methods

### Zebrafish husbandry

Wild-type AB zebrafish larvae (*Danio rerio*) were used for this study. The adult female and male zebrafish used for breeding were kept in an automated ZebTeC Benchtop Tecniplast aquarium system in 3.5 l plastic tanks and transferred to breeding tanks (Tecniplast; 1.7 l) with removable barrier and sloped egg grid the evening before breeding. Three females and two males were put in the separate chambers of the breeding tank, respectively. In the morning, the barrier between the females and males was removed and the fertilized eggs collected 1.5 h after breeding to ensure the collection of synchronous larvae. Eggs were then transferred to plastic petri dishes containing 1 × E3 medium (*Cold Spring Harbor Protocol for 60* × *E3 stock solution:* 34.8 g NaCl, 1.6 g KCl, 5.8 g CaCl_2_·2H_2_O, 9.78 g MgCl_2_·6H_2_O; dissolve in H_2_O to final volume of 2 l; adjust pH to 7.2 with NaOH) and cleaned several times to avoid fungal growth.

### Behavioral paradigms

To investigate how the hatching habitat influences consecutive habitat preferences, we used a two-choice paradigm adapted from Lucon-Xiccato et al. (2023) consisting of a habituation (see “Habituation) and a testing phase (see “Two-choice paradigms”). The same paradigm was then also used to test whether 7-day-old zebrafish larvae use spatial frequency or numerical information to choose between two numerical stimuli. Thirty zebrafish larvae (N = 30) were tested per experimental condition for both researched questions i.e., in total 360 fish were used for this study.

#### Habituation

**Fig. 1:**
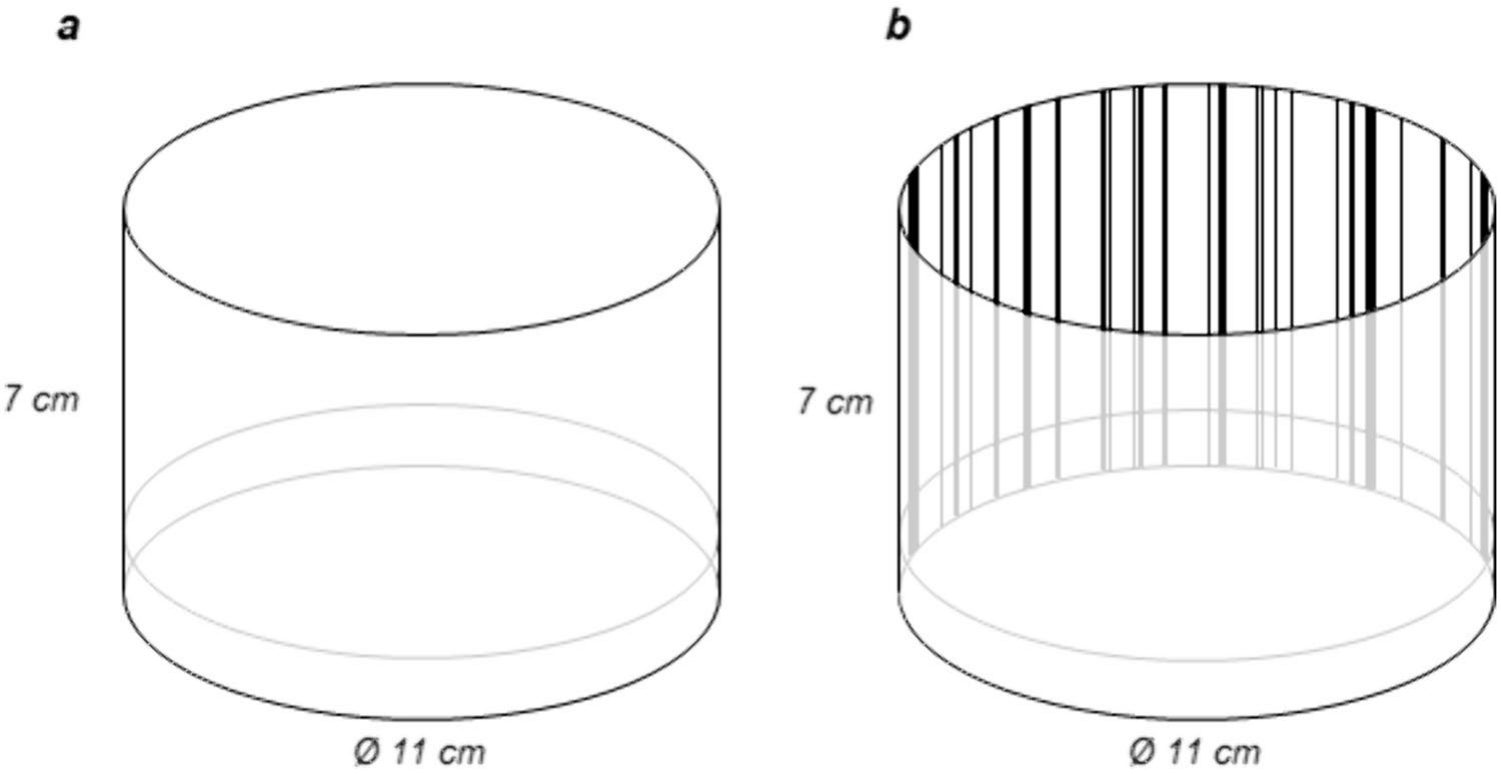
Habituation of zebrafish larvae. Glass petri dish with **a** white or **b** black bar background for habituation. The background covered the outside of the entire petri dish. The zebrafish eggs were transferred to the habituation petri dishes at 24–48 h, so the fish hatched in their respective environments. The fish were kept in the habituation petri dishes until testing. Both the white and the black bar background were used for habituation in the hatching habitat paradigm, while only the black bar background was used for habituation in the spatial frequency paradigm. *Drawings not to scale*

Cleaned zebrafish eggs were transferred to glass petri dishes (Ø 11 cm, h 1.5 cm) with a laminated paper background for habituation at 24 to 48 h. To prevent overcrowding, 50 eggs were placed per petri dish. The larvae were habituated with either a completely white background (only hatching habitat paradigm) or vertical black bars on a white background (both hatching habitat and spatial frequency paradigm) (Fig. 1; bars: w 0.2–0.4 cm, h 7 cm; distance between bars: 0.1 – 1.9 cm). Since the larvae hatched and lived in in the pre-selected environment until testing, they did not have any additional experience with other environments until testing. The E3 medium in the petri dishes was changed regularly and the larvae fed two times per day with Zebrafeed pellets (Sparos, < 100 µm) starting at 5 dpf.

#### Experimental set-up

The behavioral set-up consisted of six small white test tanks (Plexiglas: 4 mm; inside measurements: l 8 × w 5.4 × h 4.8 cm; sanded with P600 sandpaper on the inside to prevent reflection) filmed by three video cameras (Microsoft^®^ LifeCam Studio^™^, 1080p HD sensor; distance to water surface: 40 cm). Videos from these three cameras were simultaneously recorded with the software OBS Studio 29.1.2 (64 bit). A LED strip (Ledpoint lightsolutions: F52-290-60821 Honglitronic; 12V, 60 led/m, 2900-3000K, 1340 lm/m) covered with white corrugated plastic sheet (2 mm) provided uniform illumination of the tanks from above without reflectance on the water surface that would hinder filming the fish. The test tanks contained 90 ml of E3 medium, which was changed between the testing days. The stimuli were presented on the short sides of the tanks and inserted before the fish were transferred to the tank. To avoid possible side biases, the placement of the stimuli was randomized between trials.

#### Two-choice paradigms

**Fig. 2:**
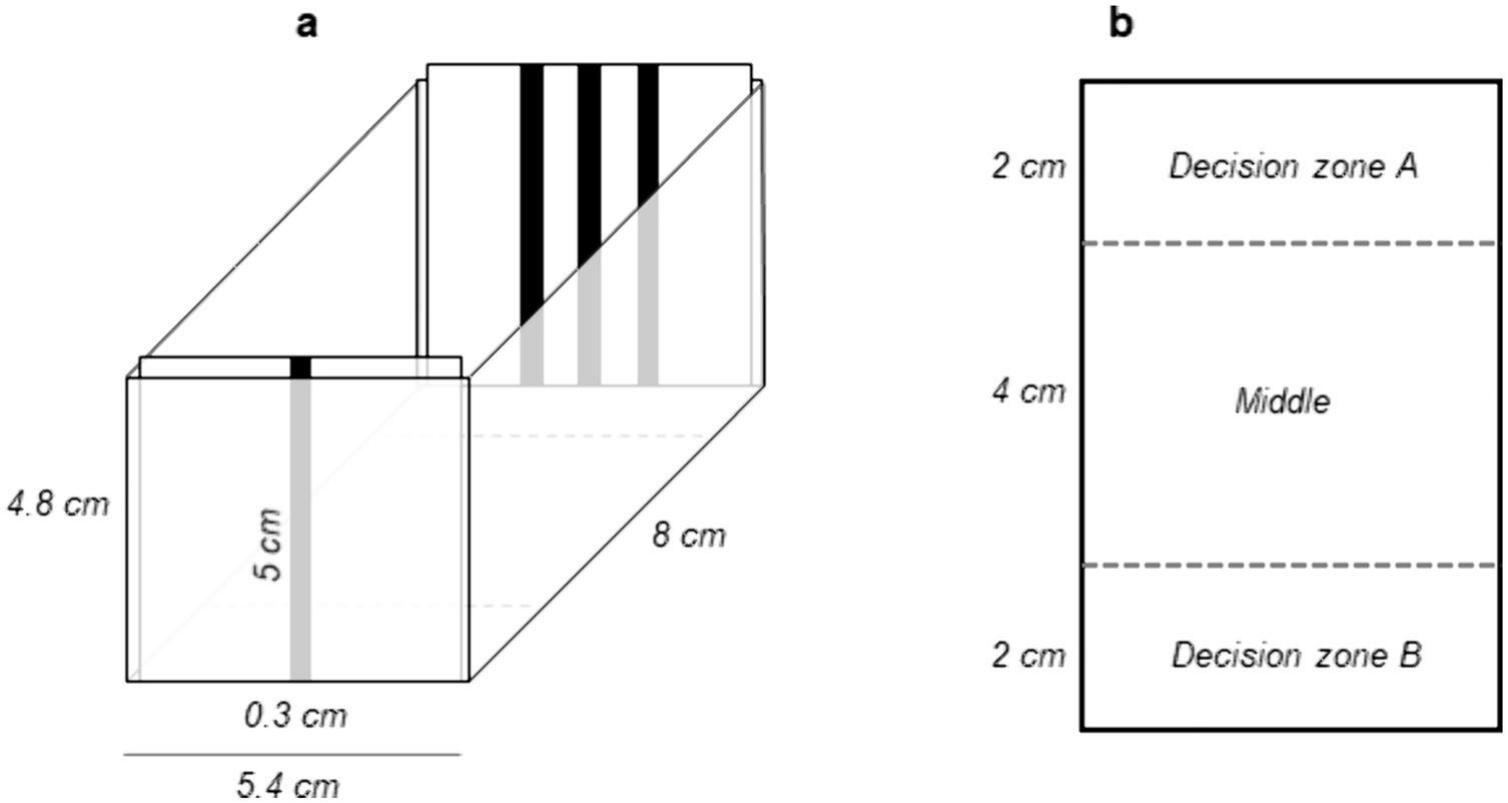
Test tank for behavioral experiments. The stimuli are inserted at the short ends of the test tank before the fish is gently placed in the middle of the tank with a pipette. **a** Side view of the test tank with 1 and 3 bar stimuli inserted. **b** Top view of the test tank with both decision zones. *Drawings not to scale*

Each fish was individually introduced into one of the test tanks and had 6 min to make a choice between two visual stimuli (Fig. 2a).

The total time spent in the respective decision zones close to the stimuli (Fig. 2) was recorded and counted as preference. The preference index was calculated by comparing the time spent close to the stimulus with the higher number of bars *vs*. the time spent close to the stimulus with the lower number of bars using the formula:

$${{Preference} \,{Index}}= \frac{{(T}_{HigherNumber}-{T}_{LowerNumber})}{({T}_{HigherNumber}+{T}_{LowerNumber})}$$

Only active fish, those that did not show freezing behavior, were used for analysis. Fish that did not enter the decision zones (i.e. did not show a preference) but showed active swimming behavior (i.e. forward movement in short swimming bouts typical for their age) within the middle section of the tank were included with a preference index of 0 (i.e. no preference) in the analysis.

To additionally assess how much time was spent in the decision zones *vs.* the middle of the tank, the preference index for the decision zones was calculated:

$${{{Preference}\, {Index}\, {Zones}}}= \frac{{{(T}_{DecisionZones}-T}_{Middle})}{({{T}_{DecisionZones}+T}_{Middle})}$$

When zebrafish larvae explore the test tank, they need time to cross the middle zone, which is twice as wide as the individual decision zones. Therefore, the larvae generally spend about an equal amount of time in the middle and the decision zones. If the fish spend, however, more time in the middle of the tank than in the decision zones, it can indicate different behaviors. The stimuli might be visually hard to distinguish and the individual might spend a lot of time swimming back and forth trying to make a choice. The larva might not make a choice at all or might stay in the middle because the middle is more attractive than the decision zones. Larvae can also show neophobia or avoidance behavior towards the new stimuli. Or the excessive cognitive load of the two stimuli could hinder the choice. We included the “Preference Index Zones” as a measure to additionally give information on whether the choice was made rather easily and/or was directed or whether other factors could have influenced it.

#### Bar stimuli

**Fig. 3:**
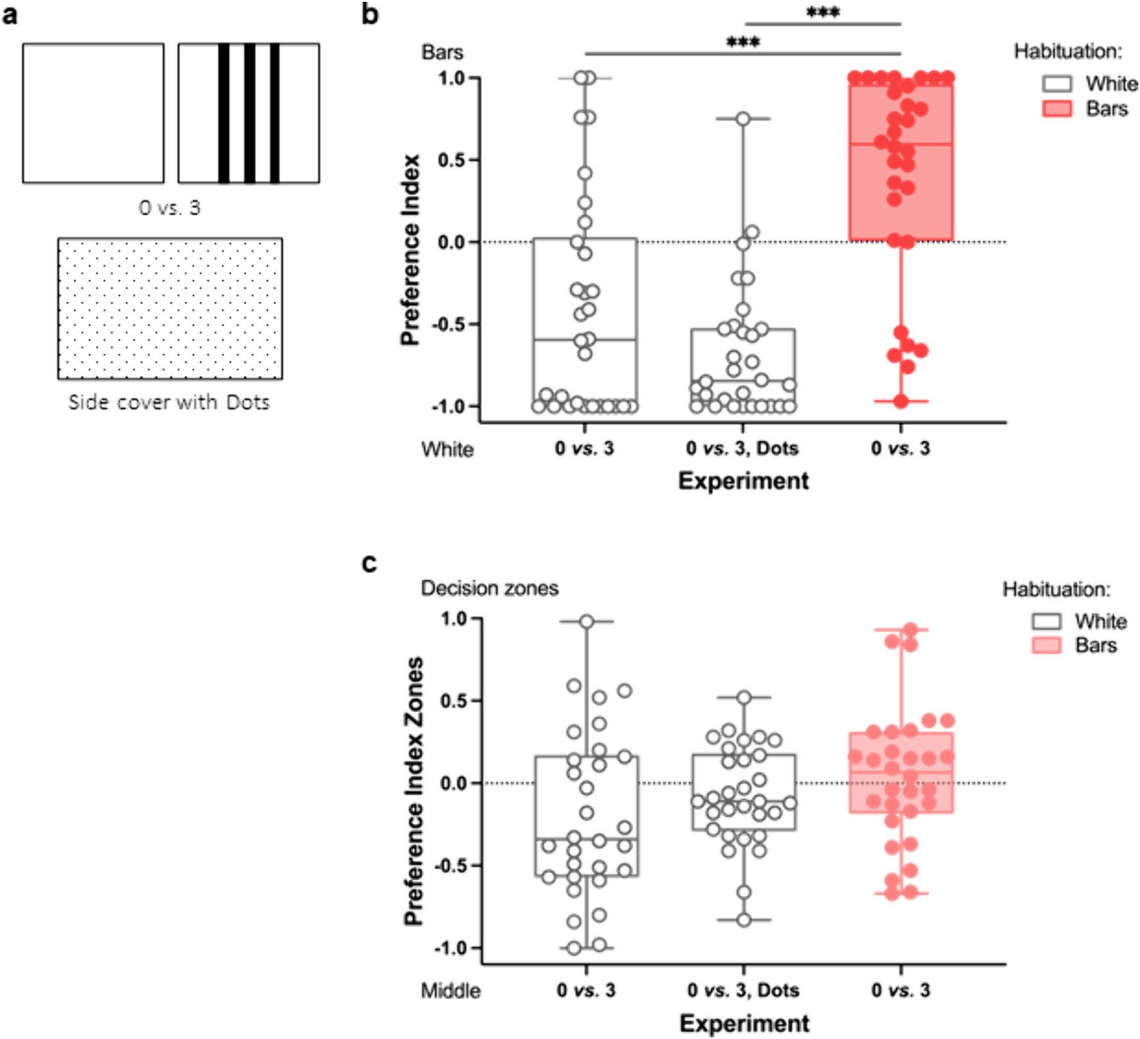
The early environment shapes the habitat preference of zebrafish. **a** White stimulus, stimulus with bars (above) and side cover for the long sides of the test tank with uniform dot pattern (below). **b** If zebrafish are habituated with a white background during the first days after hatching, they prefer the white stimulus. If they are habituated with a bar background, they prefer the bar stimulus over the white stimulus. Since the side walls of the test tank were also white, a control with a dot pattern on the side was added (0 *vs*. 3, Dots). This decreased the variability of the data and lead to a clearer preference of the fish. **c** The fish spent on average a similar amount of time in the middle and the decision zones in all three experiments. However, the variability was higher in the experiment without the dot-side covers indicating that the white walls were distracting from the white stimulus. *White – habituated with white background; Bars – habituated with bar background; Dots – test tank with side covers with dot pattern. Boxplots: Middle line: median, Box: 25th and 75th percentile, Whiskers: min, max, Dots: single data points*

**Fig. 4:**
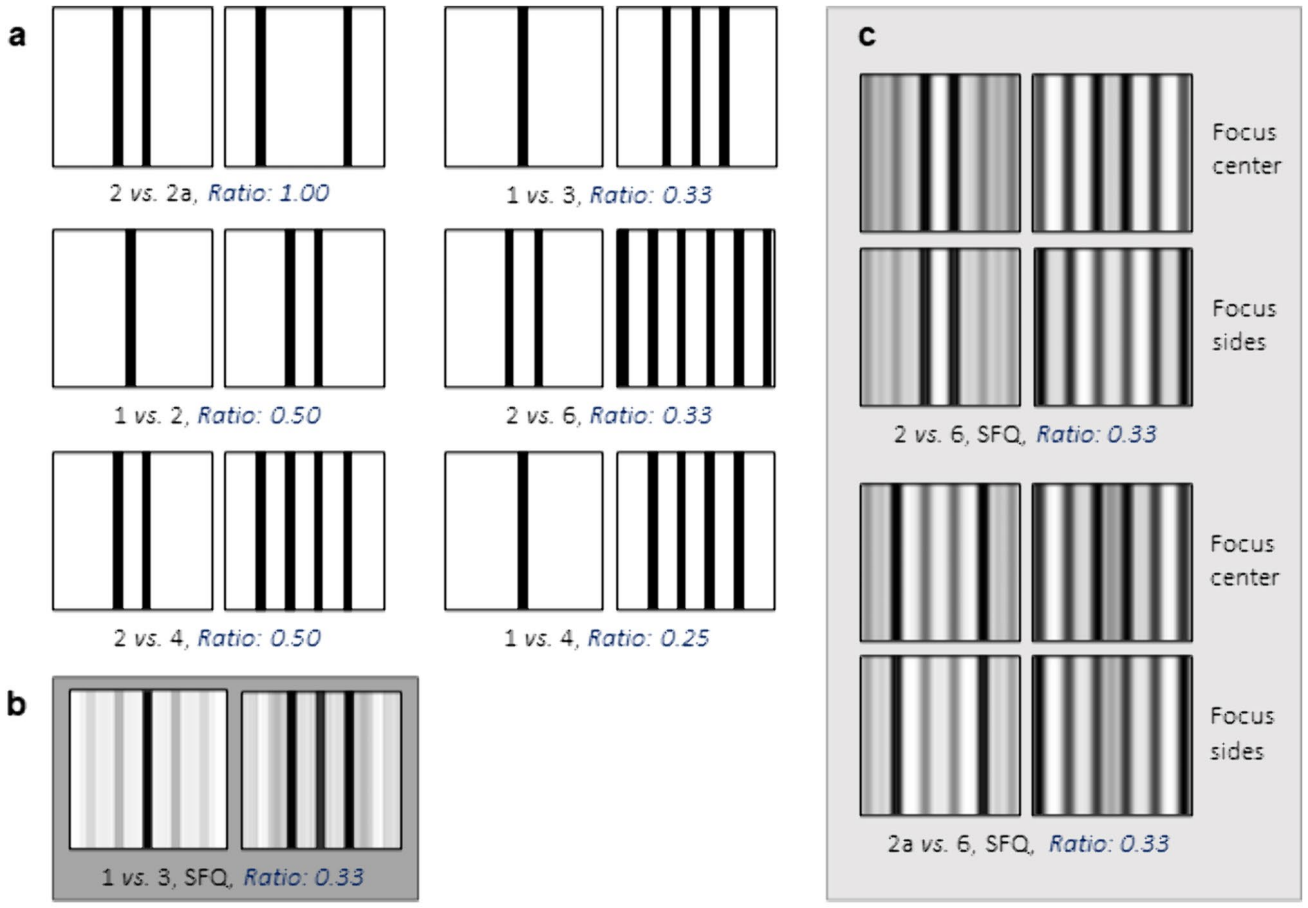
Bar stimuli for two-choice paradigm. **a** To check for the numerical preference of zebrafish, different sets of bars were used in a two-choice task. **b** Bar stimuli with 1 and 3 bars with matched spatial frequency. **c** Bar stimuli with sinusoidal grating controlled for “sharp gray phantom bars” of spatial frequency matching. *Ratio – ratio of bar numbers; the lower the ratio the easier the discrimination (Weber’s law). 2a – 2 bars apart. SFQ – stimuli with spatial frequency matched. 1 vs. 3, SFQ – spatial frequency stimuli without sinusoidal grating, 2 vs. 6, SFQ and 2a vs. 6, SFQ – spatial frequency stimuli with sinusoidal grating. Focus center – darker areas in the middle. Focus sides – darker areas on the side*

The stimuli (5 × 5 cm) and side covers (8 × 5 cm) used in the hatching habitat as well as spatial frequency paradigm consisted of laminated white paper cards (160 g/m^2^) with the black or grey patterns printed on them with a commercial printer (Koycera, TAS Kalfa 4053ci). All stimuli were designed using Microsoft PowerPoint. The black bars of the test stimuli measured w 0.3 × h 5 cm; the distance between the bars was 0.6 cm (Figs. 3a and 4). For the stimulus “2 apart” of the spatial frequency paradigm, the two outer bars of the 4-bar stimulus were used (Fig. 4a). All the dimensions were based on the evaluation of the zebrafish visual angle, using the formula:

$$V=2*\text{arctan}(\frac{S}{2*D})*(\frac{180}{\pi })$$

where V is the visual angle, S the space between the bars and D the viewing distance. The corresponding visual angles under which the bars can be observed from different places in the test tank are: 17.1° from the decision zone, 8.6° in the middle of the apparatus and 4.3° from the opposite side. Thus, all the stimuli were always distinguishable, since 5 dpf zebrafish have been shown to be able to resolve a minimum visual angle of 3.1° (Haug et al. 2010).

The spatial frequency equalization of the stimuli for the spatial frequency paradigm was performed with a custom tool implemented in “GeNEsIS” (Zanon et al. 2021), using MATLAB R2019a (The MathWorks Inc. Natick, MA, USA). To achieve this goal, we focused the analysis on the power spectrum, which is the radially averaged squared amplitude information of the fast Fourier transform of an image. This metric is commonly used in research to compare spatial frequency across visual stimuli (Adriano et al. 2021; Howard et al. 2022; Potrich et al. 2022). “GeNEsIS_frequency” allows to generate stimuli exactly matched for their spatial frequency power spectrum. The equalization is performed by calculating an average of all the power spectra of the images, which is then used to reconstruct the newly frequency-equalized stimuli. This is achieved by calculating the inverse Fourier transform from the original image phase — particular to each image — and the new average amplitude — now common to all stimuli.

In this way we could test for numerosity *vs.* spatial frequency: if the numerosity was the main information carrier for the zebrafish, it would choose the preferred numerosity over the other frequency-equalized stimulus; instead, if the frequency was used to discriminate between stimuli, the zebrafish would choose both these stimuli equally.

#### Hatching habitat paradigm

To test whether 7 dpf zebrafish larvae show a preference for the habitat they hatch in, we habituated them with either a white background or a background with black bars (Fig. 1) (see “Habituation” for details). The larvae were then tested in a two-choice paradigm with one stimulus containing no bars and the other stimulus containing three bars to investigate whether the larvae show a preference (Fig. 3a; 0 *vs.* 3). Since the walls of the test tank were also white, we added an additional control where we inserted side covers with a uniform dot pattern on both long sides of the tank to exclude attraction to the white side walls as a possible confounding factor. The dot pattern (Fig. 3a; dots: 0.5 mm, distance between dots: 2.5 mm) was selected to be very different from the habituation stimuli in order to exclude learned attraction to the pattern.

#### Spatial frequency paradigm

To investigate if 7 dpf zebrafish larvae use spatial frequency information when choosing between different numerosities, we made use of the same testing paradigm as in the hatching habitat paradigm. However, we now only used the black bar background for habituation. This ensured that the fish were attracted towards bar stimuli in general and would approach them (see also Lucon-Xiccato et al. (2023)). We first selected different bar ratios in order to find a combination that would reliably lead to numerical discrimination, so we could then test whether numerical information or spatial frequency governs the choice of the fish. We tested the combinations 2 *vs.* 2 apart (ratio 1.00, control), 1 *vs.* 2 (ratio 0.50), 1 *vs*. 3 (ratio 0.33) and 1 *vs.* 4 (ratio 0.25) as well as two similar ratios with higher bar numbers 2 *vs.* 4 (ratio 0.50) and 2 *vs.* 6 (ratio 0.33) (see Fig. 4a). Since the larvae showed a reliable preference for three bars in the combination 1 *vs.* 3 bars (ratio 0.33), we selected this ratio for our spatial frequency experiment and created two spatial frequency matched stimuli (Fig. 4b). Considering that the frequency-equalization procedure naturally introduces shadowing effects in the stimuli (to match the spatial frequency of stimuli with different numbers of bars the computation will create some “phantom bars”, mainly in the lower numerosity stimulus; Fig. 4b), in an additional condition we also slightly modified the original images to reduce this effect. This is possible by shadowing the borders of the bars to obtain a gradual passage from black to white, instead of an abrupt alternation (i.e., we used sinusoidal gratings instead of squared ones; Fig. 4c). Moreover, to better reduce the phantom bars effect after frequency equalization, we decided to use the same 1:3 ratio (0.33), but a higher number of bars (2 *vs.* 6, ratio 0.33; Fig. 4c). The sinusoidal gratings were imported into ‘GeNEsIS_frequency’ to obtain the final frequency-equalized stimuli. A control over the phantom bar intensity was also applied: even if the overall image luminosity is always balanced within frequency-equalized stimuli, the phantom bars show different intensity distributions (i.e. darker phantom bars in the center or at the borders). We balanced these two options 50–50 across both trials and pooled the data per experiment (Fig. 4c “Focus center” and “Focus sides”).

### Data analysis

Videos were analyzed blinded to the experimental condition with the VLC media player (Version: 3.0.12 Vetinari). The times the fish spent in the respective decision zone were recorded in seconds using Microsoft Excel. Data were tested for normality using the Shapiro–Wilk Test and for equality of variance using the Levene’s Test. Based on the results, the data were analyzed with a fitting parametric or non-parametric test using IBM^®^ SPSS Statistics (Version: 28.0.1.1 (14)). Paired Samples t-test and Paired Wilcoxon Signed Rank Test were used to analyze total time spent near both stimuli. Welch ANOVA and Kruskal Wallis H Test with pairwise post-hoc comparisons including Bonferroni Correction were used to compare the preference indices of different experiments. For the spatial frequency experiments *2 vs. 6, SFQ* and *2 apart vs. 6, SFQ*, Independent Samples t-Tests were conducted to determine whether the data can be pooled for the two experimental conditions “Focus center” and “Focus sides”. Graphs were plotted using GraphPad Prism version 10.2.2 for Mac OS X (Graph Pad Software, Boston, Massachusetts, USA, www.graphpad.com).

## Results

### Hatching habitat influences stimulus preference

To assess if the habitat experienced during and shortly after hatching influences the habitat preference in zebrafish, we habituated the fish with either a white or a bar background during the first days of their life until testing them at 7 dpf (Fig. 1). If the fish were habituated with the white background (*white*), they spent significantly more time near the white stimulus (Fig. 3b, 0* vs. 3, white; N* = *30; 0 bars – Mean* ± *SD: 101.4* ± *83.0 s, 3 bars – Mean* ± *SD: 43.1* ± *59.7 s; Paired Wilcoxon Signed Rank Test: Z* = *-2.606, p* = *0.009*). In contrast, if the fish were habituated with the bar background, they spent significantly more time at the bar stimulus than at the white stimulus (Fig. 3b, 0* vs. 3; N* = *30; 0 bars: 53.8* ± *63.2 s, 3 bars: 134.2* ± *93.2 s; Paired Wilcoxon Signed Rank Test: Z* = *-2.563, p* = *0.010;* Video S2: *choice for bar stimulus*).

When the fish were habituated with the bar background, they did not spent more time in the middle of the tank (Fig. 3c, 0* vs. 3 – zones; N* = *30; decision zones: 188.0* ± *74.1 s, middle: 172.0* ± *74.1 s; Paired Samples t-Test: t(29)* = *0.589, p* = *0.561*). However, if the fish were habituated with the white background, the fish spent significantly more time in the middle of the tank (Fig. 3c, 0* vs. 3, white – zones; N* = *30; decision zones: 144.5* ± *90.7 s, middle: 215.5* ± *90.7 s; Paired Samples t-Test: t(29)* = *-2.143, p* = *0.041*)*.* In this specific case, this could indicate an attraction to the white side walls of the test tank (Video S3: *example for choice of white side wall*).

Therefore, in order to exclude the possibility of the attraction to the white side walls, we conducted a control experiment where we covered the long walls of the test tank with a fine dot pattern (*Dot*; Fig. 3a “Side cover with dots”). Again, the fish spent significantly more time at the white stimulus (Fig. 3b, 0* vs. 3, white, Dot; N* = *30; 0 bars: 138.5* ± *58.5 s, 3 bars: 27.6* ± *32.8 s; Paired Wilcoxon Signed Rank Test: Z* = *-4.453, p* < *0.001*, Video S4: *choice for white stimulus*)*.* However, this time, the time the fish spent in the middle of the tank was reduced, supporting the idea that the fish in the previous experiment were attracted to the side walls as well (Fig. 3c, 0* vs. 3, white, Dot – zones; N* = *30; decision zones: 166.1* ± *54.6 s, middle: 193.9* ± *54.6 s; Paired Samples t-Test: t(29)* = *-1.391, p* = *0.175*)*.* When comparing all three experiments, the mean time spent in the middle versus the decision zones was similar (*zones: 0 vs. 3 N* = *30; 0 vs. 3, white N* = *30; 0 vs. 3, white, Dot N* = *30; Welch ANOVA: F (2, 55.466)* = *2.053, p* = *0.138*). However, the variance between the groups differed (*Levene’s Test: F(2,87)* = *4.593, p* = *0.013*). This further indicates that the control with the dot pattern was important to obtain a clearer preference by the fish habituated with the white background.

When comparing all three experiments concerning the stimulus preference, there was a significant difference between the fish that were habituated with the white background and the fish that were habituated with the bar background (Fig. 3b, *Kruskal Wallis H Test: χ*^*2*^*(2)* = *30.889, p* < *0.001; Pairwise Post-Hoc Comparison with Bonferroni Correction: 0 vs. 3 & 0 vs. 3, white: p* < *0.001; 0 vs. 3 & 0 vs. 3, white, Dot: p* < *0.001; 0 vs. 3, white & 0 vs. 3, white, Dot: p* = *0.833).* This shows that the stimulus preference depends on the first habitat the zebrafish experience.

### Numerosity: lower ratio, easier choice

To assess the numerical abilities of zebrafish larvae (7 dpf), we first habituated them with the bar background from hatching until testing in order to spark their interest in bar stimuli and then tested them with different combinations of bar stimuli (Fig. 4a).

**Fig. 5:**
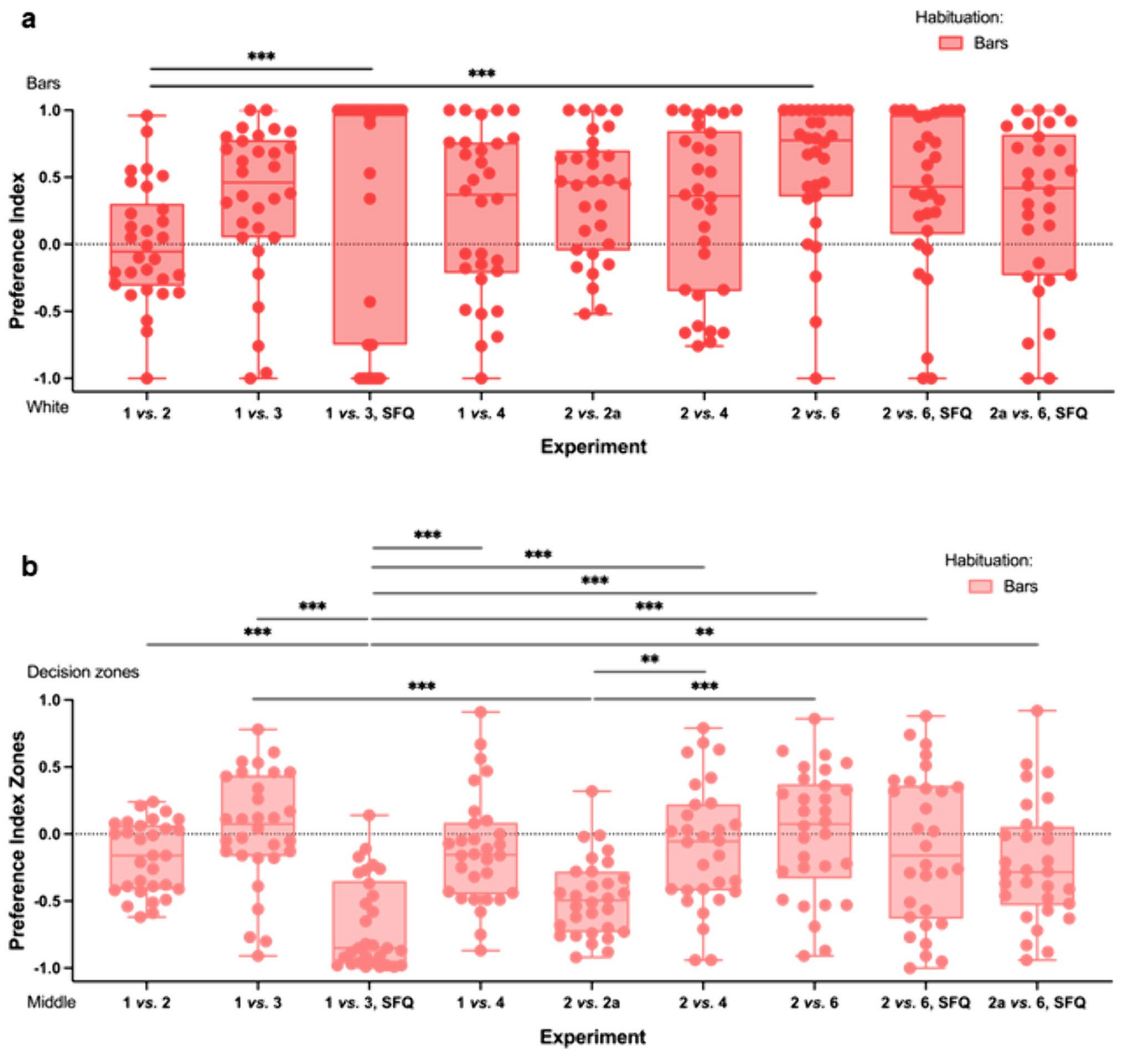
Numerosity in Zebrafish.** a** 7 dpf zebrafish larvae generally choose the higher of two numerosities in a two-choice paradigm. Their ability to choose the higher numerosity depends on the ratio of the bars. They do not, however, rely on spatial frequency, but on numerical information to make a choice (*Kruskal Wallis H Test: χ*^*2*^*(8)* = *23.759, p* = *0.003; Pairwise Post-Hoc Comparison with Bonferroni Correction: all comparisons p* = *n.s. apart from: 1 vs. 2 & 1 vs. 3, SFQ p* = *0.003 and 1 vs. 2 & 2 vs. 6 p* = *0.001*). **b** Whether the zebrafish larvae approach the stimuli can be influenced by different factors (see “Two-choice paradigms”). In experiment 1 *vs*. 3, SFQ and 2 *vs.* 2a the larvae spent significantly more time in the middle than in the other experiments. This could mean that either the choice between the stimuli was more difficult or the fish showed neophobia towards the new stimuli in 1 *vs.* 3, SFQ (*Kruskal Wallis H Test: χ*^*2*^*(8)* = *61.295, p* < *0.001; Pairwise Post-Hoc Comparison with Bonferroni Correction: all comparisons p* = *n.s. apart from: 1 vs. 3, SFQ & 1 vs. 2 p* = *0.000, 1 vs. 3, SFQ & 1 vs. 3 p* = *0.000, 1 vs. 3, SFQ & 1 vs. 4 p* = *0.000, 1 vs. 3, SFQ & 2 vs. 4 p* = *0.000, 1 vs. 3, SFQ & 2 vs. 6 p* = *0.000, 1 vs. 3, SFQ & 2 vs. 6, SFQ p* = *0.000, 1 vs. 3, SFQ & 2 apart vs. 6, SFQ p* = *0.006, 2 vs. 2 apart & 1 vs. 3 p* = *0.000, 2 vs. 2 apart & 2 vs. 4 p* = *0.039, 2 vs. 2 apart & 2 vs. 6 p* = *0.001*). *SFQ – stimuli with matched spatial frequency. Bars – habituated with bar background. 2a – 2 bars apart. Boxplots: Middle line: median, Box: 25th and 75th percentile, Whiskers: min, max, Dots: single data points*

When looking at two versus two apart (ratio 1.00), the 7 dpf zebrafish larvae showed a slight preference for the bars that were spaced apart (Fig. 5a,* 2 vs. 2 apart; N* = *30; 2 bars: 32.7* ± *32.5 s, 2 bars apart: 61.8* ± *37.4 s; Paired Wilcoxon Signed Rank Test: Z* = *− 2.898, p* < *0.004*). They spent, however, significantly more time in the middle of the tank, possibly indicating a difficult choice (Fig. 5b,* 2 vs. 2 apart – zones; N* = *30; decision zones: 94.5* ± *52.4 s, middle: 265.5* ± *52.4 s; Paired Samples t-Test: t(29)* = *-8.938, p* < *0.001*).

The zebrafish larvae (7 dpf) were not able to distinguish stimuli with a ratio of 0.50. They did not show a preference between one and two bars (Fig. 5a,* 1 vs. 2; N* = *30; 1 bar: 73.5* ± *36.1 s, 2 bars: 75.2* ± *38.8 s; Paired Samples t-Test: t(29)* = *− 0.162, p* = *0.873*, Video S5*: fish investigates both stimuli, but does not show preference*) or two and four bars (Fig. 5a,* 2 vs. 4; N* = *30; 2 bars: 65.7* ± *70.6 s, 4 bars: 97.3* ± *79.0 s; Paired Wilcoxon Signed Rank Test: Z* =− *1.306, p* < *0.191*). The fish spent, however, less time in the middle in the case of more bars (Fig. 5b,* 1 vs. 2 – zones; N* = *30; decision zones: 148.7* ± *48.1 s, middle: 211.3* ± *48.1 s; Paired Wilcoxon Singed Rank Test: Z* = *-2.692, p* = *0.007*; *2 vs. 4 – ones; N* = *30; decision zones: 163.1* ± *83.0 s, middle: 196.9* ± *83.0 s; Paired Samples t-Test: t(29)* = *− 1.115, p* = *0.274*).

When looking at the ratio of 0.33, the larvae did not spend more time in the middle than the decision zones (Fig. 5b,* 1 vs. 3 – zones; N* = *30; decision zones: 186.7* ± *76.7, middle* ± *173.3* ± *76.7; Paired Samples t-Test: t(29)* = *0.481, p* = *0.634*; *2 vs. 6 – zones; N* = *30; decision zones: 181.3* ± *84.2 s, middle: 178.7* ± *84.2 s; Paired Samples t-Test: t(29)* = *0.087, p* = *0.932*) and showed a significant preference for the stimuli with more bars (Fig. 5a,* 1 vs. 3; N* = *30; 1 bar: 55.2* ± *42.8 s, 3 bars: 131.5* ± *85.5 s; Paired Wilcoxon Singed Rank Test: Z* = *− 3.302, p* < *0.001*; *2 vs. 6; N* = *30; 2 bars: 32.9* ± *40.8 s, 6 bars: 148.4* ± *89.0; Paired Wilcoxon Signed Rank Test: Z* = *− 4.1414, p* < *0.001*).

Similarly, in the experiment with one and four bars (ratio 0.25) the zebrafish larvae did not spend more time in the middle than the decision zones (Fig. 5b,* 1vs. 4 – zones; N* = *30; decision zones: 158.8* ± *74.6 s, middle: 201.2* ± *74.6 s; Paired Samples t-Test: t(29)* = *-1.554, p* = *0.131*) and again showed a significant preference for the stimulus with four bars (Fig. 5a,* 1 vs. 4; N* = *30; 1 bar: 49.3* ± *34.3 s, 4 bars: 109.8* ± *89.2 s; Paired Wilcoxon Signed Rank Test: Z* = *− 2.448, p* = *0.014*).

Overall, these results show that zebrafish larvae (7 dpf), after early familiarization with a bar environment, spontaneously approach bar stimuli. Additionally, while facing dual-choice bar comparisons, they spontaneously spend more time close to the highest number of bars within the discrimination limit of 0.33 numerical ratio (Video S6*: choice for higher numerosity*).

### Spatial frequency vs. numerical information

To investigate whether pure numerical information governs the stimulus preference of 7-day-old zebrafish larvae, or whether spatial frequency information is confounding the choice, we further generated spatial-frequency-matched stimuli with one and three bars. Even if the stimuli were matched for spatial frequency, the zebrafish still spent more time at the stimulus with the largest numerosity (Fig. 5a, * 1 vs. 3, SFQ; N* = *30; 1 bar: 13.4* ± *26.5 s, 3 bars: 44.2* ± *59.1 s; Paired Wilcoxon Signed Rank Test: Z* = *-2.109, p* = *0.035*). However, the time spent in the middle of the test tank strongly increased and was more than five times as much as the time spent in the decision zones (Fig. 5b,* 1 vs. 3, SFQ – zones; N* = *30; decision zones: 57.6* ± *60.5 s, middle: 302.4* ± *60.5 s; Paired Wilcoxon Signed Rank Test: Z* = *− 4.742, p* < *0.001*). This increase in time in the middle seemed drastic in comparison with other experiments. Therefore, we decided to optimize the spatial-frequency stimuli and control for the sharp phantom bars since they seemed to be visually too salient.

For these adapted spatial-frequency-matched stimuli, we now used sinusoidal instead of squared gratings. We also decided to use a higher numerosity (2 or 2 bars apart vs. 6 bars) while still maintaining the ratio of 0.33, to create a more even pattern. The phantom bars in these patterns were far less pronounced and now just looked like darker areas. Still, we added an additional control for the different intensity distributions of these darker areas, with one set of stimuli having the darker areas in the center (Fig. 4c “Focus center”) and the second set of stimuli having the darker areas on the sides (Fig. 4c “Focus sides”).

For both the experiments *2 vs. 6, SFQ* and *2 apart vs. 6, SFQ*, the data of “Focus center” and “Focus sides” could be pooled since there was no significant difference between the two versions (*2 vs. 6, SFQ; Focus center: N* = *15, Focus sides: N* = *15; t(28)* = *0.137, p* = *0.892; 2 vs. 6, SFQ – zones; F. c.: N* = *15, F. s.: N* = *15; t(28)* = *− 0.137, p* = *0.892; 2 apart vs. 6, SFQ; F. c.: N* = *15, F. s.: N* = *15; t(28)* = *-0.109, p* = *0.914; 2 apart vs. 6, SFQ – zones; F. c.: N* = *15, F. s.: N* = *15; t(28)* = *0.109, p* = *0.914;* Video S7*: choice for higher numerosity of spatial frequency matched stimuli*).

The discrimination between the two bars apart and the six bars seemed harder for the fish than the choice between two bars and six bars since they spent more time in the middle of the tank (Fig. 5b,* 2 vs. 6, SFQ – zones; N* = *30; decision zones: 160.9* ± *100.9 s, middle: 199.1* ± *100.9 s; Paired Samples t-Test: t(29)* = *-1.037, p* = *0.308*; *2 apart vs. 6 – ones; N* = *30; decision zones: 140.7* ± *80.4 s, middle: 219.3* ± *80.4 s; Paired Samples t-Test: t(29)* = *-2.678, p* = *0.012*). Still, the zebrafish larvae were able to distinguish the numerosities and reliably chose the higher numerosity in both spatial-frequency-matched experiments (Fig. 5a,* 2 vs. 6, SFQ; N* = *30; 2 bars: 48.8* ± *82.5 s, 6 bars: 112.1* ± *97.9 s; Paired Wilcoxon Signed Rank Test: Z* = *-2.627, p* < *0.009*; *2 apart vs. 6; N* = *30; 2 bars apart: 40.5* ± *46.2 s, 6 bars: 100.2* ± *89.8 s; Wilcoxon Singed rank test: Z* = *-2.695, p* = *0.007*). This suggests that zebrafish larvae rely on numerosity rather than spatial frequency when assessing numerical information.

## Discussion

Our results show that zebrafish larvae (*Danio rerio*) spend more time near a stimulus that visually resembles the habitat they grew up in (Fig. 3b). This could mean that zebrafish larvae imprint on the habitat they hatch in and choose their habitat accordingly. Given that zebrafish inhabit a wide range of different habitats in the Indian subcontinent (Spence et al. 2008), this raises the question whether zebrafish larvae also choose familiar habitats over unknown habitats in the wild. It has been shown that habitat complexity as well as unpredictability can influence brain volume, cognition and behavior in different species of fish (Kotrschal and Taborsky 2010; Pollen et al. 2007; Shumway 2008). This could mean that early habitat preferences could influence later cognition and behavior in zebrafish. A field study conducted at four sites in India found that adult zebrafish exhibit different levels of aggression and shoaling preferences ranging from small, loose shoals to tightly-knit groups of hundreds of individuals depending on their habitat (Suriyampola et al. 2016). The behavioral differences seem to mainly depend on the water velocity of the water body the fish were found in. The level of predation might be higher in still and slow-moving water while fast-flowing water may constitute increased physiological costs and selective pressure on shape and body size leading to differences in social behavior and levels of aggression (Suriyampola et al. 2016). Also, in captivity, adult zebrafish have been shown to prefer habitats with water flow and vegetation over empty habitats without water flow (DePasquale et al. 2019). The preference for vegetation goes along with reduced levels of aggression and in turn increased fecundity (Carfagnini et al. 2009).

In the study of Lucon-Xiccato et al. (2023), the zebrafish larvae (7 dpf) showed a clear preference for bar stimuli after habituation with the bar background. They did not, however, show a preference for the white stimulus after habituation with a white background. This stands in contrast with our results, since we found a preference for the stimulus resembling the habituation background in both cases (Fig. 3b; Video S2 & S4). Introducing a new control and habituating the fish larvae already during hatching allowed us to improve the testing paradigm and show that the first habitat indeed influences the subsequent preference of the larvae. This is in accordance with findings that zebrafish can imprint on visual cues during their development. Adult zebrafish show a preference for similar visual characteristics when joining social groups. Fish with a dark striped phenotype show a strong preference for stimuli resembling striped fish while fish lacking stripes show a preference for stimuli with no stripes (Rosenthal and Ryan 2005). This preference for stripes or lack of stripes in conspecific does not, however, have anything to do with whether the individual has or does not have stripes itself. It is based on the social group the zebrafish has been exposed to during development (Engeszer et al. 2004; Spence and Smith 2007). Similar to our study, Engeszer et al. (2004) sorted the zebrafish into different treatment groups prior to hatching and could show that the zebrafish imprinted on the visual cues of the social group they grow up with.

What could be the mechanism behind choosing a stimulus similar to the known habitat over an unknown stimulus? In their study, Lucon-Xiccato et al. (2023) showed that when zebrafish larvae were kept in the test tank for longer than six minutes, they slowly lost the preference for the bar stimulus over time. This could mean that the zebrafish larvae choose the known stimulus as a “safe option” and show avoidance behavior toward the other stimulus and/or the rest of the tank when entering the tank. This would also support the hypothesis that black vertical bars are viewed as vegetation and that they are approached to seek cover. When time progresses, the larvae might lose their neophobia and slowly start to explore the tank.

Past studies have shown that explorative behavior can be positively influenced by environmental enrichment of the home environment in different animal species (Gatto et al. 2022; Görtz et al. 2008; Sampedro-Piquero et al. 2013; Zentall 2021). In our study, the white background could be considered a “barren” while the black bar background could be considered an “enriched” home environment. This could mean that depending on the habituation background, the zebrafish larvae could show differences in explorative behavior. In this case, spending more time exploring the middle of the test tank could be considered a sign of reduced neophobia. We did not, however, observe differences in the time spent in the middle of the test tank between the two experimental conditions (when the white side walls were controlled for). This could either mean that zebrafish larvae do not show differences in explorative behavior due to the enrichment of their home environment at 7 dpf yet or that further experiments are needed to elucidate if environmental enrichment already plays a role this early in development. The habituation for environmental enrichment studies in fish (larvae) usually lasts at least twice as long if not much longer, and larvae are tested the earliest at 13 to 14 dpf (Gatto et al. 2022, 2024; Tatemoto et al. 2021). At this age differences in recognition memory and learning due to the environmental enrichment can already be detected (Gatto et al. 2022, 2024). It will be interesting to see in future studies if those differences are already visible earlier in the development.

When processing visual information of the environment, processing speed can be essential to detect predators, food or competitors. Previous work has shown that spatial frequency information plays an important role in the rapid recognition and processing of complex visual scenes (Kauffmann et al. 2014). We therefore wanted to know if spatial frequency information plays a role in the processing of numerical information and whether zebrafish larvae rely on it or use real numerical cues to distinguish between two numerical stimuli. We first determined which numerosities of bars are easy to distinguish for 7 dpf zebrafish larvae and then created spatial-frequency-matched stimuli with different numbers of bars to determine which information is more salient for the fish larvae. When testing animals for such numerical abilities, one can either test the spontaneous choice or use operant training paradigms to teach the animal to associate a specific numerosity with a reward (Messina et al. 2022a, 2021). Although we habituated the zebrafish larvae with the bar background to draw their attention to bar stimuli, the larvae did not go through operant conditioning training and were tested on innate spontaneous preference of numerical stimuli.

The 7 dpf larvae showed almost similar numerical discrimination abilities to juvenile and adult zebrafish. Smaller ratios from 0.25 to 0.33 were quite easy for them to distinguish while ratios of 0.50 and higher were unequally harder or not possible to distinguish for these larvae (Fig. 5a). This coincides with the findings of Sheardown et al. (2022) that juvenile zebrafish at the age of 21–33 dpf reliably choose the larger group of different numerosities within ratios of 0.25 and 0.33 and that more difficult comparisons (ratio 0.67) are only feasible for older zebrafish starting from 27 dpf. Once this ability is reached, it seems to stay consistent over time since adult zebrafish have also been shown to be able to discriminate small numerosities with a ratio of up to 0.67, but failed to discriminate between numerosities with a ratio of 0.75 (Potrich et al. 2015).

When comparing our findings with the findings of Lucon-Xiccato et al. (2023), the 7-day old zebrafish larvae in their study were already able to distinguish numerosities with a ratio of 0.5, which our larvae were not. The genetic background of a zebrafish line can have an impact on the behavior of a zebrafish line and behavioral differences between different lines can be seen as early as 5 dpf (Audira et al. 2020; Burgess and Granato 2008; Crim and Lawrence 2021; van den Bos et al. 2017). In our study we used the AB wildtype line, while the authors of Lucon-Xiccato et al. (2023) used an outbred laboratory population of zebrafish that were originally purchased at a local shop. Thus, the difference in numerical ability at 7 dpf could be due to intrinsic genetic variability and/or different developmental speeds in the zebrafish lines used.

To assess whether the zebrafish larvae show a preference for a certain frequency of bars (i.e., resembling plant density), we compared two bars with different spacings. The larvae preferred the bars which were spaced further apart (i.e., at the position of the outer bars of our 4-bar stimulus). They spent, however, a substantial amount of time in the middle of the tank, which might indicate a hard choice (Fig. 5b). This result is especially interesting since we also tested 2 vs. 4 bars and did not see any preference between these two numerosities (Fig. 5a). This could indicate that, if faced with two equal numbers of bars, convex hull (the overall area occupied by a stimulus) can play a role in the choice. When the numerosities are different, numerosity seems to be more relevant than convex hull, since zebrafish larvae still choose the highest numerosity in stimuli matched for this variable (Lucon-Xiccato et al. 2023). It could also mean that more space between the bars is perceived as more space to hide between “plants”. This would be an advantage in terms of predator avoidance.

Importantly, a parameter that was not assessed by Lucon-Xiccato et al. (2023) is spatial frequency. This could be another salient cue used by animals instead of the numerical information. To investigate the use of spatial frequency in the zebrafish larvae, we first decided to use stimuli with 1 and 3 bars matched in their spatial frequency amplitude spectrum (Fig. 4b, ratio: 0.33, easily distinguishable by 7 dpf larvae).

Despite the equalized spatial frequency, the fish larvae were still able to reliably choose the higher numerosity of bars (Fig. 5a). However, since the spatial-frequency equalization had introduced some shadowing effects in the lower numerosity stimuli, we decided to add two additional experiments with sinusoidal gratings to control for the “phantom bar” visual artifacts. In this case, we kept the ratio at 0.33, but used more bars i.e. a higher numerosity (Fig. 4c). This markedly improved the visual appearance of the frequency-equalized stimuli. In addition, the new stimuli reduced the time that the fish spent in the middle of the test tank, which could either mean that they were easier to distinguish or that the fish showed less neophobia towards the spatial frequency matched stimuli with sinusoidal gratings than without them.

Again, when using the new stimuli, the zebrafish larvae chose the higher numerosity (Fig. 5a; Video S7). Since the results of Lucon-Xiccato et al. (2023) showed that zebrafish larvae do not rely on other variables such as density, cumulative surface area and convex hull to discriminate between bar stimuli with different numerosities, the preference for higher numerosity in our study suggests that zebrafish larvae use real numerical abilities rather than simpler mechanisms such as spatial frequency to distinguish between small numbers of bars. Similar results have also been shown in the archerfish (*Toxotes jaculatrix*). When testing adult archerfish for numerical discrimination, none of the continuous physical variables — including spatial frequency—were affecting the numerical preference of the fish (Potrich et al. 2022).

How could the information be processed in the brain of the zebrafish larvae? Previous work from our laboratory showed that a change in numerosity is coded in the pallium of adult zebrafish. Specifically, the immediate early genes c-fos and egr-1 are activated in the caudal part of the dorso-central division if changes in numerosity are detected by the fish (Messina et al. 2022b). This means that processing of numerosities could also take place in the pallium of the zebrafish larvae. Since spatial frequency information is processed rapidly during natural scene recognition (Kauffmann et al. 2014), that could indicate that spatial frequency information is coded further upstream in the visual pathway or that a more direct pathway could be involved. It will be interesting to determine where both numerosity and spatial frequency are coded in the larval brain in future studies.

## Supplementary Information

Below is the link to the electronic supplementary material.

Supplementary file1 (XLSX 36 KB)

Supplementary file 2: Video1_0vs3_bar.mp4. (MP4 150891 KB)

Supplementary file 3: Video2_0vs3_white.mp4. (MP4 144930 KB)

Supplementary file 4: Video3_0vs3_white_dots.mp4. (MP4 145314 KB)

Supplementary file 5: Video4_1vs2_bars.mp4. (MP4 146192 KB)

Supplementary file 6: Video5_1vs3_bars.mp4. (MP4 145671 KB)

Supplementary file 7: Video6_2vs6_SFQ.mp4. (MP4 131735 KB)

## Data Availability

The Excel file with the raw data is available as supplementary material.
